# Red Ginseng Attenuates Aβ-Induced Mitochondrial Dysfunction and Aβ-mediated Pathology in an Animal Model of Alzheimer’s Disease

**DOI:** 10.3390/ijms20123030

**Published:** 2019-06-21

**Authors:** Soo Jung Shin, Seong Gak Jeon, Jin-il Kim, Yu-on Jeong, Sujin Kim, Yong Ho Park, Seong-Kyung Lee, Hyun Ha Park, Sang Bum Hong, Sua Oh, Ji-young Hwang, Hyeon soo Kim, HyunHee Park, Yunkwon Nam, Yong Yook Lee, Jwa-Jin Kim, Sun-Hyun Park, Jong-Seok Kim, Minho Moon

**Affiliations:** 1 Department of Biochemistry, College of Medicine, Konyang University, Daejeon 35365, Korea; tlstnzz83@gmail.com (S.J.S.); jsg7394@naver.com (S.G.J.); yuon918@naver.com (Y.-o.J.); aktnfl3371@naver.com (S.K.); znf900809@naver.com (Y.H.P.); tjdrud7087@gmail.com (S.-K.L.); cubebox45@naver.com (H.H.P.); harryhong0314@gmail.com (S.B.H.); dhtndk0@naver.com (S.O.); mklop0115@naver.com (J.-y.H.); sooya1105@naver.com (H.s.K.); hyunhee16hh@gmail.com (H.P.); yunkwonnam@gmail.com (Y.N.); 2 Department of Nursing, College of Nursing, Jeju National University, Jeju-si 63243, Korea; neoreva@hanmail.net; 3 The Korean Ginseng Research Institute, Korea Ginseng Corporation, Gajeong-ro, Shinseong-dong, Yuseong-gu, Daejeon 34128, Korea; ace28@kgc.co.kr; 4 Department of Nephrology, School of Medicine, Chungnam National University, Daejeon 35015, Korea; kjj1021@naver.com; 5 R&D center for Advanced Pharmaceuticals & Evaluation, Korea Institute of toxicology, 141, Gajeong-ro, Yuseong-gu, Daejeon 34114, Korea; sunhyun.park@kitox.re.kr; 6 Myunggok Medical Research Institute, College of Medicine, Konyang University, Daejeon 35365, Korea; jskim7488@konyang.ac.kr

**Keywords:** Alzheimer’s disease, amyloid beta, mitochondria, red ginseng, 5XFAD mice

## Abstract

Alzheimer’s disease (AD) is the most common neurodegenerative disease and is characterized by neurodegeneration and cognitive deficits. Amyloid beta (Aβ) peptide is known to be a major cause of AD pathogenesis. However, recent studies have clarified that mitochondrial deficiency is also a mediator or trigger for AD development. Interestingly, red ginseng (RG) has been demonstrated to have beneficial effects on AD pathology. However, there is no evidence showing whether RG extract (RGE) can inhibit the mitochondrial deficit-mediated pathology in the experimental models of AD. The effects of RGE on Aβ-mediated mitochondrial deficiency were investigated in both HT22 mouse hippocampal neuronal cells and the brains of 5XFAD Aβ-overexpressing transgenic mice. To examine whether RGE can affect mitochondria-related pathology, we used immunohistostaining to study the effects of RGE on Aβ accumulation, neuroinflammation, neurodegeneration, and impaired adult hippocampal neurogenesis in hippocampal formation of 5XFAD mice. In vitro and in vivo findings indicated that RGE significantly improves Aβ-induced mitochondrial pathology. In addition, RGE significantly ameliorated AD-related pathology, such as Aβ deposition, gliosis, and neuronal loss, and deficits in adult hippocampal neurogenesis in brains with AD. Our results suggest that RGE may be a mitochondria-targeting agent for the treatment of AD.

## 1. Introduction

Alzheimer’s disease (AD), the most common cause of dementia, has a reported incidence of 50%–56% among dementia patients, and is a highly prevalent neurodegenerative disorder [1,2]. One of the pathological hallmarks of AD is the aggregation of amyloid beta (Aβ) in the brain [3,4]. The Aβ cascade hypothesis indicates that its progenitor peptide, amyloid precursor protein (APP), is sequentially cleaved by β-secretase and γ-secretase consecutively to produce Aβ [5,6]. These Aβ peptides induce neuroinflammation, neuronal loss, and alteration of adult neurogenesis [7,8]. A number of recent studies have demonstrated that mitochondria may mediate Aβ-induced pathology and initiate the AD pathogenesis [9,10].

Mitochondria are responsible for multiple functions, such as energy production, regulation of metabolism, cellular signaling modulation, calcium buffering, and neuronal functions [11]. In addition, mitochondrial dysfunctions including aberrant mitochondrial gene expression resulting in apoptosis [2,12,13], abnormal mitochondrial membrane potential, vulnerability to oxygen glucose deprivation, and reactive oxygen species production [14] are directly associated with AD-related pathology. In cortical neurons, Aβ activates the significant mitochondrial fission [15]. Furthermore, isolated rat brain mitochondria in an AD rat model exhibited decreased cytochrome c oxidase (COX) and tricarboxylic acid cycle enzyme activities [16]. When mitochondria are damaged, ATP production is decreased in neurons, and Aβ-induced oxidative stress negatively affects neurotransmission, synaptic functions, and cognitive functions such as memory in AD patients [12,17]. The mitochondrial deficits can mediate or trigger the AD pathogenesis [9]. It has been proposed that when mitochondrial dysfunction occurs, accumulation of Aβ increases, which might result in a vicious cycle that contributes to the onset and progression of AD. In this series of processes, mitochondrial dysfunction may contribute as the primary or secondary pathogenic factor [12]. Alterations in mitochondrial protein composition induced by Aβ accumulation may provide evidence for a reciprocal relationship between AD and mitochondrial dysfunction [18]. In summary, mitochondrial cascade activity modulates Aβ-induced AD pathology. Thus, preventing mitochondria from becoming a mediator of Aβ toxicity can be a key to AD therapy.

A number of studies have focused on natural compounds to identify potential drug candidates for AD treatment [19]. *Panax ginseng* Meyer (PG) is known to have beneficial effects in the treatment and prevention of neurodegenerative diseases such as Parkinson’s disease (PD) and AD [20]. In particular, red ginseng (RG), a processed form of PG obtained by steaming and drying, is well known to be a therapeutic material for various conditions, and many previous studies have demonstrated the various beneficial effects of RG on biological functions [20]. RG has been shown to improve cognitive functions of healthy male participants in a randomized controlled trial study [21]. Moreover, RG extract (RGE) has been shown to improve cognitive function by reducing inflammatory activity in the hippocampus of aged mice [22]. In addition, RG attenuates the learning and memory deficits in young rats with hippocampal lesions and aged rats, and these effects may be mediated by the effects of RG on hippocampal formation [23].

Given that cognitive enhancement is considered as a key target for AD treatment [24], the memory-enhancing effect of RG might be beneficial for AD patients. Consistently, the cognitive enhancing effects of adjuvant RG treatment with conventional anti-dementia medications has been clinically confirmed in patients with AD [25,26]. Furthermore, administration of RG results in an improvement in the frontal lobe function of AD patients, implying the potential for a substantive medicinal effect of RG [27].

Although previous studies have reported the protective effect of RG on mitochondrial dysfunction in the arachidonic acid and iron-induced cytotoxicity models [28] as well as adult hippocampal neurogenesis in the 1-methyl-4-phenyl-1,2,3,6-tetrahydropyridine-induced mice model of PD [29], studies that have directly assessed the effects of RG on adult hippocampal neurogenesis and mitochondrial dysfunction in AD are difficult to find. More importantly, as mentioned above, the importance of the role of mitochondrial dysfunction in AD is increasing. Thus, mitochondrial dysfunction might be a therapeutic target for the treatment of AD. In addition, there is no histological study examining the effect of RG on AD pathologies induced by Aβ. These gaps in the literature prompted us to examine the effects of RG on mitochondrial dysfunction and Aβ-mediated pathologies. Here, we report that RGE attenuated mitochondrial dysfunction and Aβ-mediated pathologies including Aβ deposition, gliosis, and neuronal loss, and decreased adult hippocampal neurogenesis in 5XFAD mice, an animal model of AD.

## 2. Results

### 2.1. Cytotoxicity Evaluation of RGE in Hippocampal Neurons

We examined the cytotoxicity of RGE in the HT22 hippocampal neuronal cell line. The results obtained using the 3-(4,5-dimethylthiazol-2-yl)-2,5-diphenyltetrazolium bromide (MTT) assay indicated that incubation with RGE at concentrations of 1, 10, 100, 500, and 1000 μg/mL for 24 h did not induce significant neurotoxicity (Figure S1A). However, cytotoxicity was observed after incubation with RGE for 48 h at concentrations of 500 and 1000 μg/mL (Figure S1B). Therefore, we performed the subsequent experiments using RGE concentrations of 1–100 μg/mL for 24 h, which did not cause neurotoxicity in the hippocampal cells.

### 2.2. RGE Prevents Aβ-Induced Mitochondrial Dysfunction in HT22 Cells

**Figure 1:**
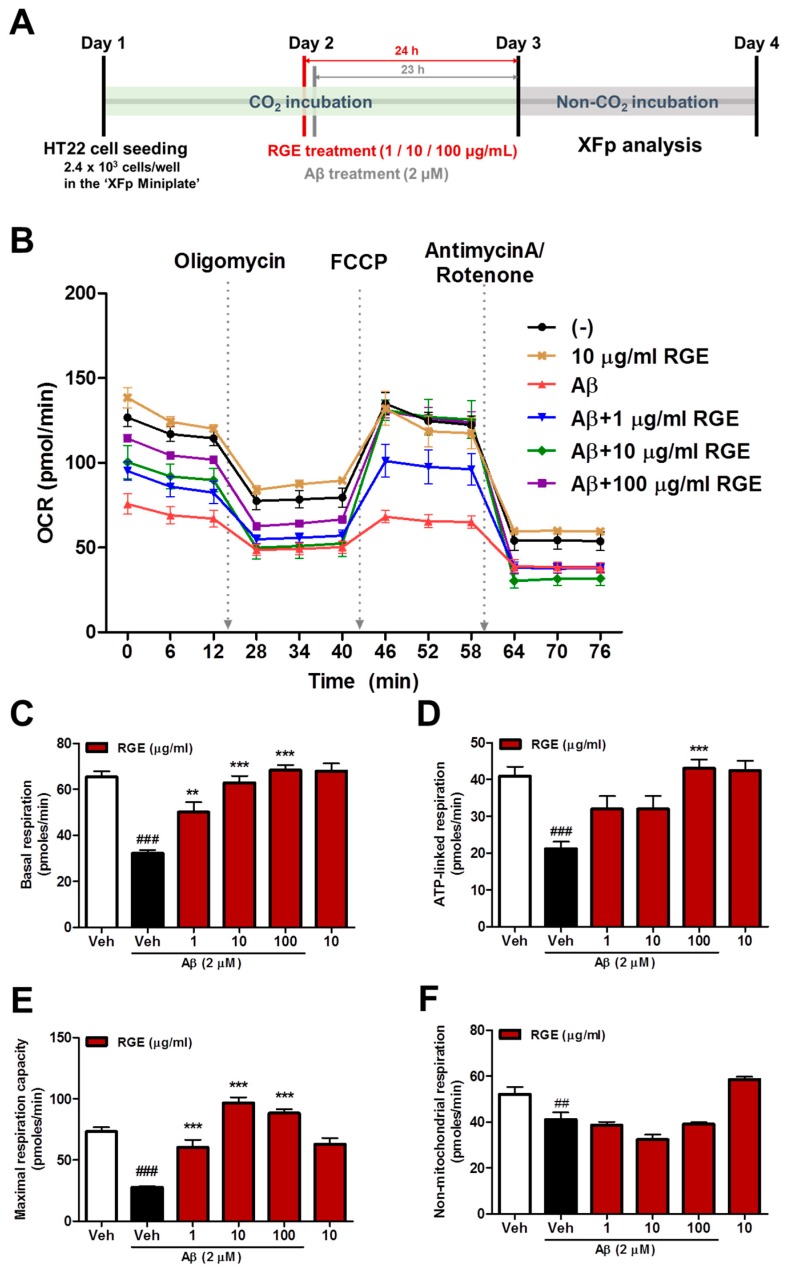
Red ginseng extract (RGE) prevents amyloid beta (Aβ)-mediated mitochondrial dysfunction in HT22 cells. (**A**) Outline of the experimental design and timeline for mitochondrial respiration assessment with Seahorse XFp analysis. (**B**) The Seahorse assay using the XFp analyzer was performed to measure the oxygen consumption rate (OCR) after treatment with Aβ (2 μM) and Aβ + RGE (1, 10, and 100 μg/mL). Basal respiration (**C**), ATP-linked reparation (**D**), maximal respiration capacity (**E**), and non-mitochondrial respiration (**F**) were measured by analyzing OCR values by adding oligomycin (1 μM), FCCP (1 μM), rotenone (0.5 μM), and antimycin A (0.5 μM). Data are presented as mean ± SEM. ^##^ *p* < 0.01 and ^###^ *p* < 0.001: vehicle-treated cells (white bar) versus Aβ-treated cells (black bar). ^**^ *p* < 0.01 and ^***^ *p* < 0.001: Aβ-treated cells versus Aβ + RGE-treated cells (red bar).

Although the protective effect of ginseng on mitochondrial deficits is well known [30,31], there is no evidence for the effect of RGE on Aβ-induced mitochondrial dysfunction. Thus, to determine the effects of RGE on Aβ-induced mitochondrial deficits, cultured HT22 cells were treated with Aβ (2 μM) and/or RGE (1, 10, and 100 μg/mL) and the oxygen consumption rate (OCR) was measured using the Seahorse XFp analyzer (Figure 1B). Aβ-treated HT22 cells showed a significant decrease in basal respiration resulting from mitochondrial proton leakage and ATP demand (Figure 1C). The RGE treatment dose-dependently rescued the basal respiration impairment caused by Aβ (Figure 1C). ATP-linked respiration, which is determined on the basis of the decreased level of OCR due to the addition of ATP synthetase inhibitor oligomycin (1 μM), was also significantly reduced by Aβ treatment (Figure 1D). However, treatment with RGE at a dose of 100 μg/mL restored ATP-linked respiration to a similar level as that in the control group (Figure 1D). Maximum respiratory capacity as determined by the maximum OCR level mimics the physiological energy demand by the addition of the carbonyl cyanide 4-(trifluoromethoxy)phenylhydrazone (FCCP) (2 μM), a mitochondrial uncoupler. Treatment with RGE dose-dependently improved the maximum respiratory capacity impaired by Aβ (Figure 1E). The persistent OCR level noted after blocking the hydrogen ion gradient between the intermembrane space and mitochondrial matrix by addition of inhibitors of complex I (rotenone, 0.5 μM) and complex III (antimycin A, 0.5 μM) represents non-mitochondrial respiration sustained by a subset of cellular enzymes. However, the Aβ-induced reduction in non-mitochondrial respiration was not restored by RGE treatment (Figure 1F). Overall, these results indicate that RGE could alleviate Aβ-induced mitochondrial deficit in hippocampal neurons.

### 2.3. RGE Alleviates Abnormal Mitochondrial Dynamics in Aβ-Overexpressing Transgenic Mice

**Figure 2:**
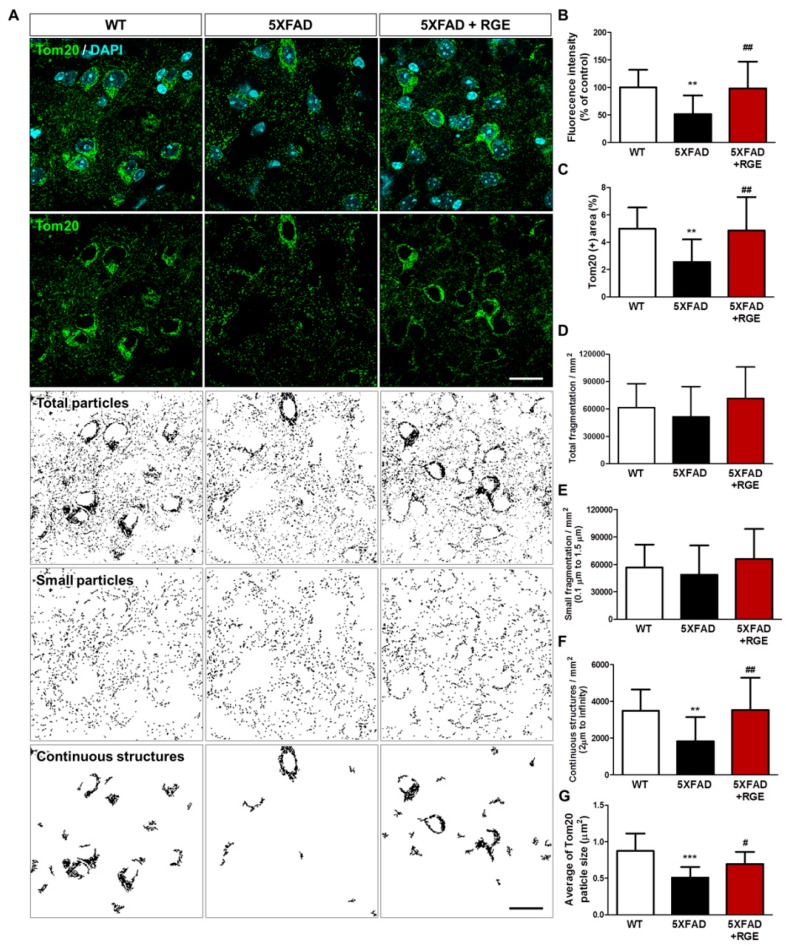
Effects of RGE on changes in mitochondrial dynamics caused by Aβ accumulation in the subiculum of 5XFAD mice. (**A**) Immunohistochemical staining for translocases of mitochondrial outer membrane 20 (Tom20) was performed to evaluate the mitochondrial dynamics (scale bar = 20 μm). (**B**) The quantification results of Tom20 fluorescence intensity were normalized to the values for vehicle-treated wild-type (WT) mice. (**C**) Tom20 (+) area was quantified and plotted as a percentage. Total fragmentation (**D**), small fragmentation (**E**), continuous structures (**F**), and average Tom20 particle size (**G**) were quantified by setting the immunoreactivity area of Tom20 to a threshold by mitochondrial structure size to identify the fusion and fission of mitochondria. Data are presented as mean ± SEM. ** *p* < 0.01 and *** *p* < 0.001: vehicle-treated WT mice (white bar) versus vehicle-treated 5XFAD mice (black bar). # *p* < 0.05 and ## *p* < 0.01: vehicle-treated 5XFAD mice versus Aβ + RGE-treated 5XFAD mice (red bar).

The imbalance in mitochondrial dynamics, in which fusion and fission proteins coexist, is a seminal event leading to aberrant mitochondrial fragmentation and neurodegeneration [32,33]. To investigate the effect of RGE treatment on disruption of mitochondrial dynamics, we performed immunohistochemical staining with antibodies against anti-translocases of mitochondrial outer membrane 20 (Tom20), which is a marker for the mitochondrial outer membrane, in hippocampal formations, including the subiculum, of 5XFAD mice. For quantitative and morphological analysis of mitochondrial dynamics, Tom20 (+) areas were reconstructed by threshold and size distribution using Image J software (Figure 2A). The immunoreactive areas and fluorescence intensity of Tom20 were remarkably diminished in 5XFAD mice compared with those in wild-type (WT) mice, whereas RGE administration significantly suppressed this diminishment (Figure 2B,C). In the reconstructed results for Tom20 in the subiculum, there was no significant difference between the three groups in terms of the total fragmentation and the small fragmentation (Figure 2D,E). The continuous structure and average size were significantly higher in the subiculum of 5XFAD mice in comparison with those in WT mice. However, the 5XFAD mice that received RGE exhibited improvement in continuous structure and average size in the subiculum of vehicle-treated 5XFAD (Figure 2F,G). These results indicate the protective effect of RGE on the imbalance in mitochondrial fusion and fission during Aβ-induced mitochondrial dysfunction in the brain with AD.

### 2.4. RGE Reduces Aβ Deposits in the Subiculum of 5XFAD Mice

**Figure 3:**
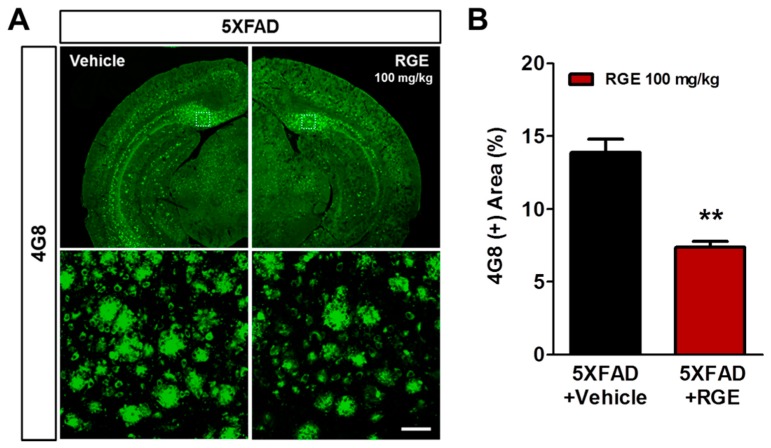
Effects of RGE on Aβ accumulation in the subiculum of 5XFAD mice. (**A**) Immunohistochemical analysis of Aβ burden was performed with the 4G8 antibody in the subiculum of 5XFAD mice (scale bar = 100 μm). (**B**) 4G8 (+) areas were significantly reduced in the subiculum in RGE-treated 5XFAD mice compared with those in vehicle-treated 5XFAD mice. Data are presented as mean ± SEM. ** *p* < 0.01.

Mitochondrial dysfunction is associated with Aβ accumulation [9]. In the early stage of AD, Aβ accumulation and neuronal loss were increased in the subiculum [34,35]. Therefore, we investigated whether anti-mitochondrial RGE might affect Aβ deposition in the subiculum of 5XFAD mice. To examine Aβ accumulation, brain sections were immunohistochemically stained with 4G8 antibody (Figure 3A). In these assessments, the 4G8 (+) area in the subiculum of 5XFAD mice was significantly reduced by RGE treatment (Figure 3B). These results suggest that RGE with anti-mitochondrial activity significantly reduces the Aβ burden in the brain of Aβ-overexpressing transgenic mice.

### 2.5. RGE Attenuates Neuroinflammation and Neuronal Death in the Subiculum of 5XFAD Mice

**Figure 4:**
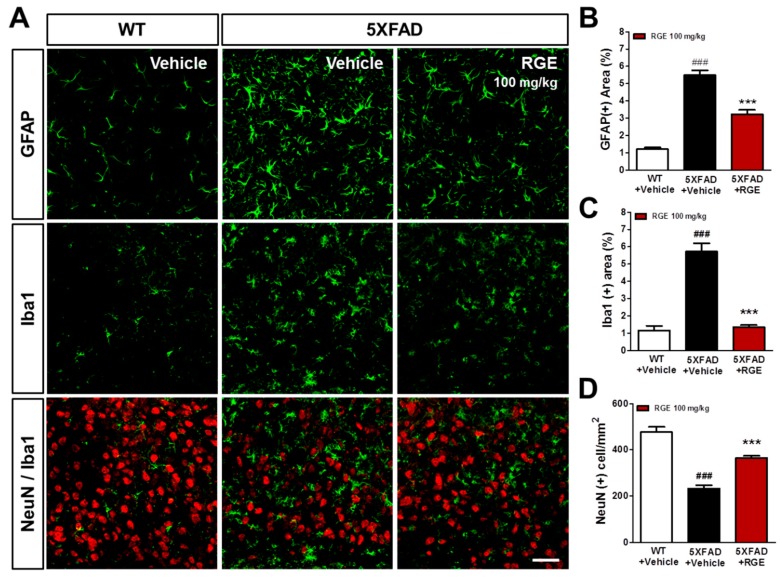
Effects of RGE on neuroinflammation in the subiculum of 5XFAD mice. (**A**) Immunofluorescent staining was conducted with the markers of microglia (ionized calcium-binding adapter molecule 1, Iba-1), astrocytes (glial fibrillary acidic protein, GFAP), and neurons (neuronal nuclei, NeuN) in the subiculum (scale bar = 100 μm). (**B**) The significantly greater GFAP-positive areas in 5XFAD mice compared to those in WT mice were significantly reduced by RGE administration. (**C**) The significantly greater Iba1 (+) areas in 5XFAD mice compared to those in WT mice were significantly reduced by RGE administration. (**D**) The number of NeuN (+) cells per area was significantly reduced in 5XFAD mice compared to those in WT mice and significantly improved by RGE administration. Data are presented as mean ± SEM. *** *p* < 0.001: vehicle-treated WT mice (white bar) versus vehicle-treated 5XFAD mice (gray bar). ^###^ *p* < 0.001: vehicle-treated 5XFAD mice versus Aβ + RGE-treated 5XFAD mice (black bar).

Over-activation of astrocytes and microglia coexisting with Aβ aggregates are indices of neuroinflammation and one of the pathological features of the AD brain [7,36,37,38]. In addition, mitochondrial deficit causes a vicious cycle leading to neuronal death mediating neuroinflammation [9,39]. Therefore, we studied whether the mitochondrial protection afforded by RGE may affect the neuroinflammation and neurodegeneration in the subiculum of Aβ-overexpressing transgenic mice. Immunohistochemical staining with antibodies against GFAP (glial fibrillary acidic protein for astrocyte), Iba-1 (ionized calcium-binding adapter molecule 1 for microglia), and NeuN (neuronal nuclei, neuron) was performed in the subiculum of 5XFAD mice (Figure 4A). A significant increase in the Iba-1 (+) and GFAP (+) areas with morphological activation was observed in 5XFAD mice, and these increases were significantly alleviated by RGE administration (Figure 4B,C). Neuronal loss accompanied by microglial activation in 5XFAD mice was also protected by RGE administration (Figure 4D). These results suggest that the administration of RGE inhibits the gliosis and neuronal loss in Aβ-overexpressing transgenic mice, suggesting that anti-mitochondrial RGE could contribute to attenuation of neuroinflammation as well as neurodegeneration in the AD brain.

### 2.6. RGE Improves Impaired Hippocampal Adult Neurogenesis in 5XFAD Mice

**Figure 5:**
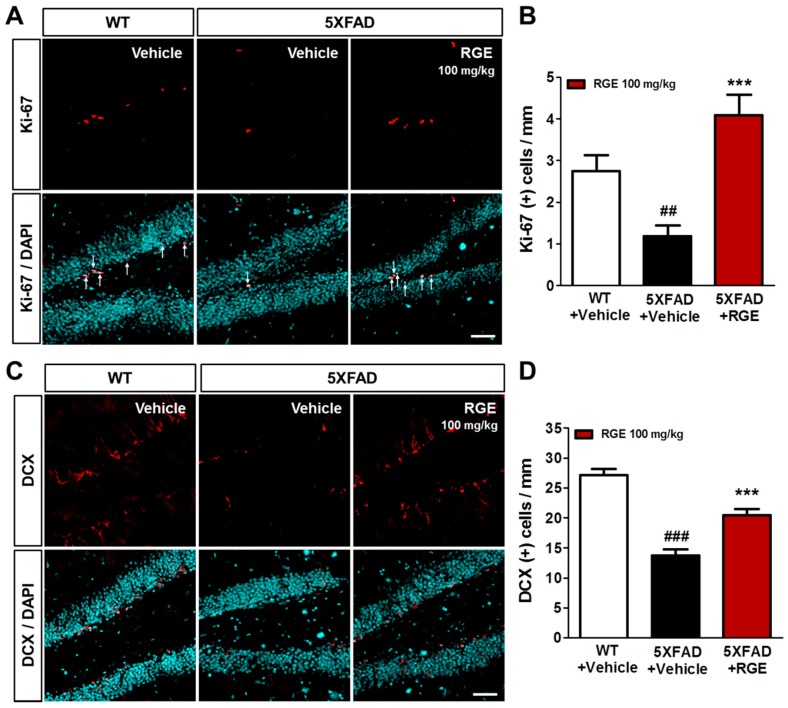
Effects of RGE on adult hippocampal neurogenesis in 5XFAD mice. (**A**) Immunofluorescent staining was performed in the subgranular zone (SGZ) of the dentate gyrus (DG) by using Ki-67, a marker of proliferation. White arrows indicate Ki-67 (+) cells (scale bar = 50 μm). (**B**) The number of Ki-67 (+) cells per area was significantly reduced in 5XFAD mice compared to that in WT mice and significantly increased by RGE administration. (**C**) Immunofluorescent staining was performed in the SGZ of DG by using doublecortin (DCX), a marker of neuronal precursors (scale bar = 50 μm). (**D**) The number of DCX (+) cells per area was significantly reduced in 5XFAD mice compared to that in WT mice and significantly increased by RGE administration. Data are presented as mean ± SEM. *** *p* < 0.001: vehicle-treated WT mice (white bar) versus vehicle-treated 5XFAD mice (gray bar). ^##^ *p* < 0.01 and ^###^ *p* < 0.001: vehicle-treated 5XFAD mice versus Aβ + RGE-treated 5XFAD mice (black bar).

Adult hippocampal neurogenesis, known to play a significant role in the regulation of cognitive function [40,41,42,43], is altered in the AD brains of animal models and patients [8,44,45]. In addition, mitochondria are known to play a crucial role in adult neurogenesis [46,47]. Therefore, restoration of impaired hippocampal neurogenesis has been suggested as a therapeutic strategy for AD [8,48]. Although the administration of RG has been reported to improve cognitive function in AD patients [25,26,49], no evidence has been provided for the effect of RGE on hippocampal neurogenesis. To investigate the effect of RGE on the impaired adult hippocampal neurogenesis in 5XFAD mice, immunofluorescent staining was performed with the Ki-67 antibody, a marker of proliferation, and the doublecortin (DCX) antibody, a marker of neuronal precursors in the subgranular zone (SGZ) of the hippocampus (Figure 5A,B). The number of Ki-67 (+) and DCX (+) cells per SGZ length significantly reduced in the 5XFAD mice in comparison with the vehicle-treated WT of 5XFAD mice. However, RGE administration restored the reductions in the number of Ki-67 (+) and DCX (+) cells in the SGZ of 5XFAD mice (Figure 5C,D). To our knowledge, these results are the first histological evidence that RGE treatment improves the impaired adult hippocampal neurogenesis in brains with AD.

## 3. Discussion

**Figure 6:**
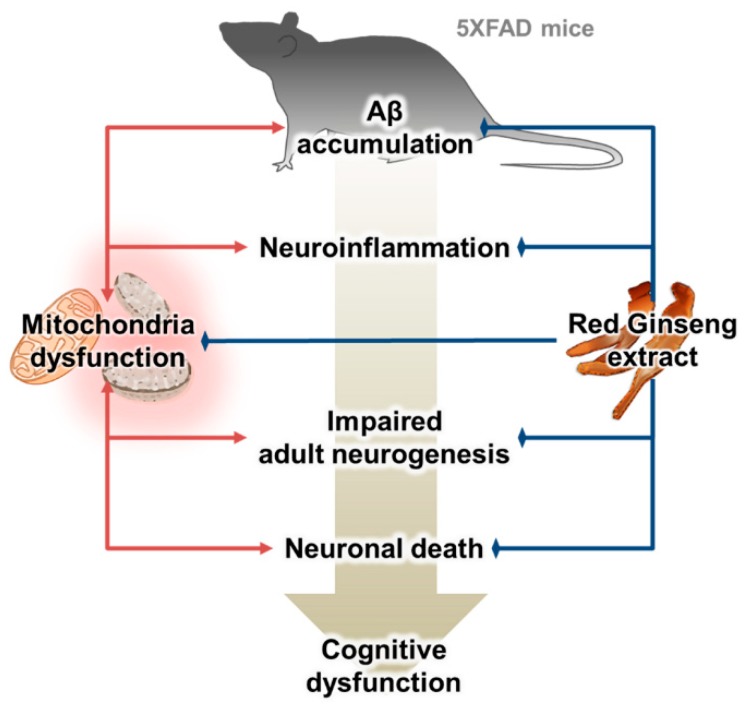
Schematic diagram of the effect of RGE on Alzheimer’s disease (AD) pathology modulated by Aβ-induced mitochondrial dysfunction.

Since the roles of Aβ in the pathogenesis of AD have been extensively investigated, the importance of the roles of mitochondrial dysfunction in AD is increasing. Although a number of studies have reported the beneficial effects of RG on Aβ-mediated pathologies, there were no studies examining its effects on mitochondrial dysfunction in AD. Moreover, there is no histological study examining the effect of RG on the AD pathologies induced by Aβ. Therefore, we studied the effects of RGE on mitochondrial dysfunction and Aβ-mediated pathologies. Here, we report that RGE ameliorates mitochondrial dysfunction and Aβ-mediated pathologies, including Aβ deposition, gliosis, and neuronal loss, and decreased adult hippocampal neurogenesis in 5XFAD mice, an animal model of AD (Figure 6).

In both the in vitro and in vivo studies, treatment with RGE significantly restored the impaired mitochondrial respiratory capacity and protected against the imbalance in mitochondrial fusion and fission during Aβ-induced mitochondrial dysfunction (Figure 1 and Figure 2). Previous studies have suggested an association between Aβ and mitochondrial functions in AD. Aβ not only attacks key mitochondrial enzymes such as cytochrome c oxidase (complex IV), pyruvate dehydrogenase, and α-ketoglutarate, but also damages mtDNA, leading to mitochondrial dysfunction [16,50]. Enhancement of mitochondrial homeostasis has been reported to counteract the proteotoxicity and aggregation of Aβ [51]. Since damaged mitochondria are hypothesized to be a factor responsible for Aβ generation as well as the result of Aβ and APP toxicity [12], maintenance of mitochondrial functions might be advantageous in reducing Aβ levels and Aβ-induced pathologies. Moreover, the balance in the mitochondrial dynamics of neuronal cell division and development is important, indicating that unbalanced mitochondrial dynamics causes several diseases [52]. When mitochondrial deficits occur due to Aβ, the mitochondrial fusion and fission are altered indicating imbalance of mitochondrial dynamics [53].

Although no studies have evaluated the efficacy of whole RG in mitochondrial dysfunction of AD brain, there are several reports on the beneficial effects of RG and its related components in mitochondria. It has been reported that RGE inhibits mitochondrial damage and apoptotic cell death through the activation of AMPK pathway in arachidonic acid + iron-induced mitochondrial dysfunction [28]. PG root has been traditionally consumed as a decoction for longevity [54]. The ethanol extract of PG has been reported to be involved in the regulation of mitochondrial bioenergetics in cardiomyoblast with antioxidant effect [55]. The major pharmacologically active ingredients of ginseng are ginsenosides and gintonin [56,57]. Ginsenosides are further classified into protopanaxadiol (e.g., Rb1) or protopanaxatriol (e.g., Rg1) [58]. The ginsenoside Rg1, one of components of RG, shows protective effects against Aβ-related mitochondrial dysfunction. In particular, Rg1 plays regulatory roles in maintaining mitochondrial membrane potential, normal ATP production, COX activity, ROS production, and caspase-3 activity [59]. The ginsenoside Rg3 beneficially affects cardiac mitochondrial population quality, and the ginsenoside Rd reduces mitochondrial permeability transition and release of cytochrome C in the spinal cord [60,61]. Especially, Rg3 has been reported to not only increase the expression of antioxidant-related proteins such as heme oxygenase-1 and nuclear factor erythroid 2–related factor-2 but also contribute to antiaging by preventing ultraviolet-induced mitochondrial dysfunction in human dermal fibroblast [62]. In addition, PG polysaccharide improved mitochondrial dysfunction and metabolism with antioxidant effect by increased creatine kinase activity [63]. These findings suggest that various components, such as ginsenosides, in the RGE may have positive effects on mitochondrial dysfunction. Notwithstanding, interpretation should be addressed with caution since present study did not examine the effects of RGE on pro-apoptotic proteins and ROS. An empirical study using RG should be followed to confirm the molecular mechanisms involved in efficacy of whole RG.

As expected, a significant reduction in the 4G8 (+) area was observed in 5XFAD mice treated with RGE, indicating that RGE prevents Aβ accumulation (Figure 3). Several studies have reported that ginseng extract and its major component, ginsenosides, could be involved in the amyloidogenic mechanism to alleviate abnormal Aβ homeostasis [64,65,66]. Moreover, gintonin, a ginseng-derived G protein-coupled lysophosphatidic acid receptor ligand, has been recently identified [57]. Recent research shows that ginsenosides downregulate β-secretase activity, reducing amyloidogenic processes [65,67]. Furthermore, gintonin promotes sAPPα release, and lessens Aβ_1-40_-induced cytotoxicity [68]. As mentioned previously, ginseng-derived ingredients show extensive and diverse efficacy for treatment of AD. Remarkably, the amount of ginsenoside contents of RG is not only 1.7 times higher than that of PG, but RG has also been reported to contain several ginsenosides not found in PG [69]. Therefore, it is suggested that RG may have a stronger pharmacological potential than PG.

In the present study, we observed a significant reduction in glial activation and gliosis after treatment with RGE (Figure 4). Previous studies have consistently reported that the major active ingredients, such as ginsenosides and gintonin, inhibit microglial activation and Aβ-mediated neuroinflammation [68,70,71]. These anti-inflammatory effects could be considered in relation to the protective effects on mitochondrial dysfunction. Neuroinflammation is also one of the factors that cause synaptic and neuronal damage in AD. Interestingly, neuroinflammation has been known to be associated with mitochondrial deficits [39,72]. Mitochondrial deficits caused by Aβ can lead to cell death, and the microglial activators subsequently released by degenerating neurons might contribute to neuroinflammation [9,73]. In this context, the pharmacological action of RGE on neuroinflammation may be related to its pharmacological action mechanism for mitochondrial function. Thus, RG can reduce cell death by inhibiting mitochondrial deficits caused by Aβ, suggesting that this may ultimately mitigate neuroinflammation.

Mitochondria have been known to regulate adult neurogenesis and cognitive function [46,47]. In the brain with AD, the balance of mitochondrial fission and fusion is impaired [53]. This imbalance in mitochondrial dynamics may contribute to altered neurogenesis in AD. Therefore, if the mitochondrial dynamics can be normally maintained, the impaired neurogenesis in AD may also be restored [47]. In our study, RGE ameliorated the mitochondrial dysfunction as well as the adult hippocampal neurogenesis (Figure 5). In particular, 5XFAD mice that received RGE showed a significantly increased number of Ki-67 (+) cells than did the WT mice. Although the mechanisms by which RGE affects adult neurogenesis have not been fully elucidated, many of the previously mentioned factors, including the maintenance of mitochondrial dynamics, may have improved adult hippocampal neurogenesis in 5XFAD mice. Based on these evidences and our results, it is reasonable to assume that RGE does not simply enhance the mitochondrial respiratory function, but also prevents Aβ deposition and Aβ-related pathologies directly or indirectly by mediating the recovery of mitochondrial functions. Adult neurogenesis is an important factor directly related to cognitive function [74]. Adult neurogenesis and cognitive function decrease together as aging progresses, and drugs that improve adult neurogenesis may be used as an adjunct to AD therapy [75]. Although we used 3.5-month-aged mice showing no significant memory deficits [76], a previous study has reported that enhancement of mitochondrial function increased adult hippocampal neurogenesis [47]. Thus, it can be speculated that RGE may also prevent the cognitive decline in AD.

Currently, the FDA is focused on two pharmacologic modalities for AD therapy: acetylcholinesterase (AChE) inhibitors and N-methyl-D-aspartate receptor antagonists [77]. The AD drugs currently on the market could delay the progression of the disease, and most are AChE inhibitors [78]. However, long-term treatment with pharmacological control can cause a variety of side effects [79,80]. Above all, they are not used as curative treatment that restores neurocellular function, but rather allopathic treatment that alleviates the symptoms [81]. Although there is no comprehensive AD treatment that can be used clinically as a substitute for previous drugs yet, it is expected that the use of complementary drugs that are directly involved in AD pathology along with the existing drugs will produce partial therapeutic effects as well as mitigating symptoms. As an alternative to this approach, there are reports of strategies for AD treatment. In particular, herbal medicine using drugs such as PG are attracting attention in neurodegenerative diseases, particularly AD treatments [82,83]. PG has long been used to alleviate symptoms related to many diseases, particularly aging and memory loss [84]. RG not only has anti-oxidative and anti-aging effects [85,86], but also has, as seen in our experiments, protective-effects on the neurons from Aβ, which is considered the main cause of AD. Moreover, it can be speculated that RG preserves the neuronal cell by protecting the mitochondrial function from Aβ and by increasing the level of adult neurogenesis, which is likely to be a treatment approach that is fundamental to AD pathology. Therefore, the ability of RG to protect the mitochondria can be used to prevent AD and can even serve as a potential therapeutic approach. However, these interpretations should be made with caution, since there are different types of interaction between natural products and their compounds [87], implying that effects of RG and its single components might be substantially different.

As is well known, mitochondria are the heart of ATP production. Recent studies have shown that ATP acts as a biological hydrotrope and inhibits the self-aggregation of Aβ_42_ peptides [88]. ATP production in Aβ-induced mitochondrial dysfunction is reduced in AD. Aβ causes mitochondrial dysfunction, which causes deficits in the mitochondrial respiratory chain, resulting in a decrease in ATP production. [89]. Therefore, protecting mitochondrial ATP production by addressing the ATP inhibition caused by peptide aggregation is critical for the prevention and treatment of AD. This reduction in mitochondrial deficits can be a key contributor to AD prevention and treatment, leading to the conclusion that RG, which protects mitochondrial function, can be a potential therapeutic agent for AD. Aforementioned above, this protective action on the mitochondrial function of RGE ultimately might reduce neuroinflammation, thereby protecting neuronal cells and promoting adult neurogenesis. Thus, our conclusion is that RGE can contribute to AD therapy by protecting neuronal cells. The neuroprotective effect of PG [90] and the enhancement of cognitive function through neuroprotection of RG-induced compound K [91], can support our conclusions. However, our study focuses on the effect of RGE on AD by studying its effects not on the neuronal cell itself, but on the mitochondria within the neuronal cell, with the difference that total extract was used. There have been a number of studies using RGE isolates, including ginsenosides regarding their effects on AD, but our knowledge of the effects of total extract of RG are limited [20]. The influence of the pharmacological components of RGE and their interactions are still not fully understood. Thus, our experiments using the total extract can provide data reflecting more variables related to drug efficacy in comparison with other tests using only isolated specific efficacy. Therefore, this study implies that RGE itself, not its isolated components, acts as a mitochondrial guardian. Nevertheless, we only evaluated the toxicity of the RGE using an in vitro method, implying that further toxicological evaluation with an in vivo model could provide evidence for safety in clinical use.

## 4. Materials and Methods

### 4.1. Preparation and Characterization of RGE

Commercial Korea RGE (Hongsamjung, Cheong-Kwan-Jang) made from with *Panax ginseng* Meyer (6-year-old Korean origin) was purchased. In order to suitably improve the sticky semi-solid formulation of commercial RGE for the experiment, commercial RGE dissolved in distilled water, freeze-dried in a freeze dryer (Operon, Gyeonggi, Republic of Korea), and ground into fine powder. The powder was used in the subsequent experiments. The RGE powder was analyzed to determine its arginine-fructose-glucose (AFG), acidic polysaccharide (AP), and ginsenoside content. The analysis of AFG [92] and AP [93,94] was based on the respective methods. The analysis of ginsenosides was performed as previously described [95]. For analysis of ginsenosides, 0.5 g of the RGE powder was shaken vigorously after the addition of 10 mL of 70% MeOH. Extraction was performed in an ultrasonicator (60 Hz, Wiseclean; Daihan Scientific, Seoul, Republic of Korea) for 30 min. After ultrasonic extraction, centrifugal separation (Legand Mach 1.6R; Thermo, Frankfurt, Germany) was performed for 10 min at 3000 rpm. The resulting supernatant solution was filtered (0.2 mm, Acrodisk; Gelman Sciences, Ann Arbor, MI, USA) and injected into the UPLC-photo diode array (PDA) system (Waters Co., Milford, MA, USA). In the next step, instrumental analysis was performed by a Waters ACQUITY UPLC system (Waters, Millford, MA, USA) composed of a binary solvent manager, sample manager, and PDA detector. Chromatographic separation was accomplished on an ACQUITY BEH C18 column (2.1 × 50 mm, 1.7 um; Waters). The column temperature was 40 °C. The binary gradient elution system consisted of deionized water (A) and acetonitrile (B). UPLC gradient conditions were as follows: 0.5–14.5 min (15%–30% B), 14.5–15.5 min (30%–32% B), 15.5–16.5 min (32%–40% B), 16.5–17.0 min (40%–55% B), 17.0–21.0 min (55%–90% B), 21–25 min (90%–15% B), and 25–27 min (15% B). The flow rate was set at 0.6 mL/min, and the sample injection volume was 2.0 μL. The 11 ginsenosides were detected by PDA at 203 nm. For analysis of AP, we used the carbazole-sulfuric acid method, which is known to measure the amount of uronic acid that constitutes hexuronic acid or polyuronide contained in plants. Briefly, prepared and mixed 0.5 mL of 50 mg/mL RGE solution, 0.25 mL carbazole in 0.1% ethanol and 3 mL concentrated H_2_SO_4_. Then, the mixed solution was heated in 80 °C water for 5 min and it was cooled for 15 min in room temperature. The results of analysis for ginsenosides, AFG, and AP contained in RGE are shown in Table 1.

### 4.2. Culture of HT22 Cell Line

The mouse hippocampal neuronal HT22 cell line was incubated with Dulbecco’s modified Eagle’s medium (DMEM; WELGENE, Gyeongsan-si, Korea) consisting of 10% fetal bovine serum (FBS; GenDEPOT, Katy, TX, USA) and 100 U/mL of penicillin-streptomycin (Gibco, Waltham, MA, USA) at 37 °C under 5% CO_2_.

### 4.3. Cell Viability Assay

The MTT (3-(4,5-dimethylthiazol-2-yl)-2,5-diphenyltetrazolium bromide; Sigma-Aldrich, St. Louis, MO, USA) assay was used to measure cell viability via mitochondrial reductase. HT22 cells were seeded into a 96-well plate at a density of 5 × 10^3^ cells/well in DMEM medium with 10% FBS and incubated for 24 h. Then, the culture medium was treated with RGE (1, 10, 100, 500, and 1000 μg/mL) for 24 or 48 h. MTT solution was added to each well to a final concentration of 0.5 mg/mL and incubated for 2 h at 37 °C. After removing the culture medium containing MTT, dimethyl sulfoxide was added and incubated to dissolve the reduced formazan crystals at 37 °C for 1 h. The absorbance was measured at 540 nm.

### 4.4. Measurements of OCR

HT22 cells were seeded in XFp Miniplate at a density of 2.4 × 10^3^ cells/well. Twenty-four hours later, the cells were treated with RGE (1, 10 or 100 μg/mL) and Aβ_1-42_ (2 μM; Bachem, Bubendorf, Switzerland) sequentially, followed by XFp analysis (Figure 1A). OCR was measured according to the manufacturer’s protocol using a Seahorse XFp Cell Mito Stress Test Kit (Agilent Technologies, Santa Clara, CA, USA) with Seahorse XFp Analyzer (Agilent Technologies). The Seahorse XF base medium used during the XFp analysis procedure was reconstructed with d-glucose (4.5 mg/mL), l-glutamine (584 μg/mL), and sodium pyruvate (110 μg/mL). The final well concentrations of loaded compound per port of the sensor cartridge were designated as follows: Port A (1 μM oligomycin), Port B (1 μM FCCP), and Port C (0.5 μM rotenone/antimycin A). The OCR in pmol/min was measured every 6 min; the ports of the sensor cartridge were opened one by one in alphabetical order at the end of every three measurements, and the compounds were released to each well of the XFp Miniplate. The analysis results obtained by triplicate repetition were automatically calculated and integrated by the XF Cell Mito Stress Test Report Generator software (version 2.6.0.31).

### 4.5. Animals and Administration

5XFAD mice that expressed five familial AD mutations at the human presenilin 1 (*PSEN1*) gene (M146 and L286) and human *APP* gene (K670N/M671L, V717I, and I716V) were obtained from Jackson Laboratory (Bar Harbor, ME, USA). The littermates obtained through crossing of male 5XFAD mice and female B6SJL/F1 mice (Jackson Laboratory) were classified into wild-type (WT) mice and 5XFAD mice by genotyping and prepared for experiments (*APP* forward: 5′-AGG ACT GAC CAC TCG ACC AG-3′, *APP* reverse: 5′-CGG GGG TCT AGT TCT GCA T-3′, *PSEN1* forward: 5′-AAT AGA GAA CGG CAG GAG CA-3′, *PSEN1* reverse: 5′-GCC ATG AGG GCA CTA ATC AT-3′). Female 5XFAD and WT mice at 3.5 months of age were used in the present study. The RGE was reconstituted in saline before administration. The extracts were orally administered at a dose of 100 mg/kg every other day for 4 weeks (Figure S2). The dose was determined based on previous studies examining the effects of RGE using animal models [96,97,98]. The experiment was conducted in three groups: (1) WT+vehicle (*n* = 6), (2) 5XFAD+vehicle (*n* = 7), and (3) 5XFAD+RGE (*n* = 6). The animals were maintained in accordance with the National Institutes of Health (NIH) guide for the care and the use of Laboratory Animals (NIH Publications No. 8023, revised 1978). In addition, the experiment was conducted and reviewed under the supervision of the Institutional Animal Care and Use Committee at Konyang University (project identification code: P-19-03-A-01, date: 17 February 2019).

### 4.6. Preparation of Brain Tissue

The mice were sacrificed the day after the final injection of RGE. The animals were cardiac-perfused with 4% paraformaldehyde (PFA) in 0.05 M phosphate-buffered saline (PBS). The brains were removed and post-fixed in 4% PFA for 20 h at 4 °C. To preserve them in the frozen state, the brains were submerged in a solution containing 30% sucrose in 0.05 M PBS for 3 days. They were sectioned at a 30-μm thickness in the coronal plane with a CM1850 cryostat (Leica Biosystems, Wetzlar, Germany) and stored in cryoprotectant solution (25% ethylene glycol, 25% glycerol in 0.05 M phosphate buffer) at 4 °C until use.

### 4.7. Immunofluorescence Labeling

For histological staining and analysis, three brain tissues obtained at 210–240-μm intervals were anatomized from each mouse from the region between −2.6 and −4.3 mm to the bregma [99]. Primary antibodies including mouse Tom20 antibody (1:200), mouse 4G8 antibody (1:2000), mouse anti-NeuN antibody (1:100), rat anti-GFAP antibody (1:200), goat anti-Iba-1 antibody (1:500), goat anti-DCX antibody (1:50), and rabbit anti-Ki-67 antibody (1:200) were prepared at their individual dilution ratios in PBS containing 0.3% Triton X-100 and 0.5 mg/mL BSA. The brain sections were incubated with the primary antibody overnight at 4 °C. Next, the tissues were washed three times for 5 min with PBS and incubated with the secondary antibody for 1 h at room temperature. Secondary antibodies, including goat Alexa Fluor^®^ 488 conjugated anti-mouse antibody, donkey Alexa Fluor^®^ 488-conjugated anti-rat antibody, donkey Alexa Fluor^®^ 488-conjugated anti-goat antibody, goat Alexa Fluor^®^ 488-conjugated anti-rabbit antibody, and donkey Alexa Fluor^®^ 594-conjugated anti-goat antibody, were prepared at 1:500 dilution in PBS containing 0.3% Triton X-100. After washing three times with PBS, the brain tissues were placed on SuperFrost Plus^™^ Adhesion slides and then cover-slipped with Fluoroshield^™^ with 4′,6-diamidino-2-phenylindole (DAPI).

### 4.8. Image Acquisition and Analysis

Images of all histochemical samples were obtained with a Zeiss LSM 700 (Carl Zeiss AG, Oberkochen, German), and the images were analyzed using ImageJ software (NIH, Bethesda, MD, USA). For the analysis of 4G8, GFAP, and Iba-1 immunoreactivity, the area fractions of immune-positive signals in the brain tissues were quantified. The number of NeuN-positive cells was quantified as the number of immune-responsive cells per mm^2^ area of tissue. The number of Ki-67 or DCX-positive cells was quantified as the number of immune-positive cells per length of the SGZ in the dentate gyrus (DG). Quantification of Tom20 immunoactivity was performed by determining the percentage of the Tom20 (+) area and fluorescence intensity using ImageJ software (NIH, Bethesda, MD, USA). For the mitochondrial dynamics, only the Tom20 (+) area was set to a threshold, after which small fragments (0.1–1.5 μm) and continuous structure (2 μm to infinity) were sorted according to the Tom20 (+) area size and counted per area. The total fragmentation (0 μm to infinity) of the Tom20 (+) area was counted and the particle size was averaged.

### 4.9. Statistical Analysis

All statistical analyses were performed using GraphPad Prism 5.0 software (GraphPad Software, Inc., La Jolla, CA, USA). Data are presented as mean ± standard error of the mean (SEM). The Kolmogorov–Smirnov test was used for normality verification and the F-test was used for analysis of variance between groups. An unpaired *t*-test with Welch’s correction was used for comparisons between two groups. For statistical analysis between three or more groups, one-way analysis of variance test with Tukey’s post hoc test was used for the group passed normality, and the Kruskal–Wallis test with Dunn’s post hoc test was used for the case that the normality was not satisfied. *p*-value of < 0.05 indicated statistical significance.

## 5. Conclusions

RGE has been suggested to have protective effects on mitochondrial dysfunctions, Aβ deposition, and Aβ-related pathologies, implying that RGE might be a potential agent for substantial treatment of AD. The specific molecular mechanism by which the mitochondrial deficits lead to lesions and the processes and parts of the mitochondria that RGE influences are not yet clear. Further studies revealing the specific mechanism underlying the beneficial effects of RGE are needed. Therefore, we propose a study to completely identify the kinds of pharmacological components contained in RGE and the molecular interrelationships between them. Moreover, it would be a good approach to assess how these molecular interrelationships change the physiological mechanisms and interact. By establishing the physiological mechanism of RGE through a molecular biology perspective, insights into the potential of using RG as an AD therapeutic agent can also be obtained. Aside from this suggestion, based on the studies reporting different composition and efficacy among different forms of ginseng product [92,100], it would be worthwhile to evaluate the effects of various forms on AD-related pathologies as well as mitochondrial dysfunction.

## Figures and Tables

**Table 1:** Characterization of red ginseng extract.

Ginsenoside (mg/g)											AFG (mg/g)	AP (mg/g)
Rb1	Rb2	Rc	Rd	Re	Rf	Rg1	Rg2s	Rg3r	Rg3s	Rh1		
6.23	2.45	2.94	1.27	0.93	1.37	0.64	1.78	1.77	3.50	1.68	5.58	98.46

AFG: arginine-fructose-glucose, AP: acidic polysaccharide.

## Supplementary Materials

Supplementary materials can be found at https://www.mdpi.com/1422-0067/20/12/3030/s1.

## Abbreviations

Aβ	amyloid beta
AChE	acetylcholinesterase
AD	Alzheimer’s disease
AFG	arginine-fructose-glucose
AP	acidic polysaccharide
APP	amyloid precursor protein
COX	cytochrome c oxidase
DAPI	4′,6-diamidino-2-phenylindole
DCX	doublecortin
DG	dentate gyrus
DMEM	Dulbecco’s modified Eagle’s medium
GFAP	glial fibrillary acidic protein
Iba-1	ionized calcium-binding adapter molecule 1
MTT	3-(4,5-dimethylthiazol-2-yl)-2,5-diphenyltetrazolium bromide
NeuN	neuronal nuclei
OCR	oxygen consumption rate
PBS	phosphate-buffered saline
PD	Parkinson’s disease
PFA	paraformaldehyde
PG	*Panax ginseng* Meyer
RG	red ginseng
