# Supplementary material for: Red Ginseng Attenuates Aβ-Induced Mitochondrial Dysfunction and Aβ-mediated Pathology in an Animal Model of Alzheimer’s Disease
Source: Int J Mol Sci. 2019 Jun 21;20(12):3030. doi: 10.3390/ijms20123030 (PMC6627470; doi:10.3390/ijms20123030)
Supplement: Supplementary file 1 [file ijms-20-03030-s001.pdf]

Supplementary Materials

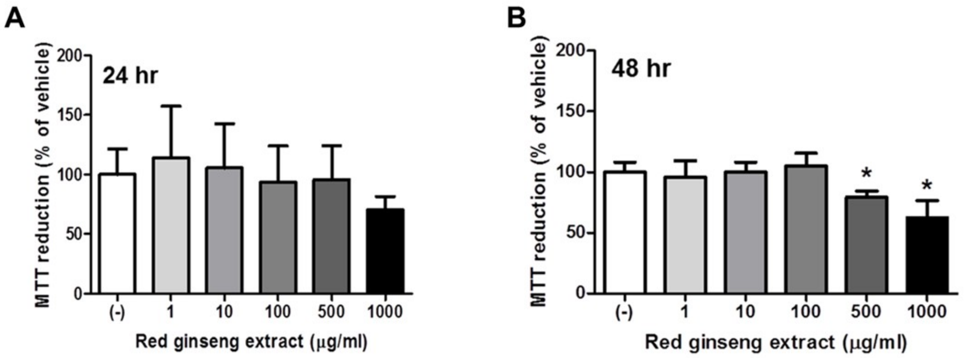

Supplementary Figure 1. Cytotoxicity of RGE to the HT22 cell line. HT22 hippocampal neuronal cells were treated with various concentrations of RGE (1, 10, 100, 500, and 1000 μg/ml) for 24 h (A) and 48 h (B). $^{*}p < 0.05$ compared with the vehicle-treated group.

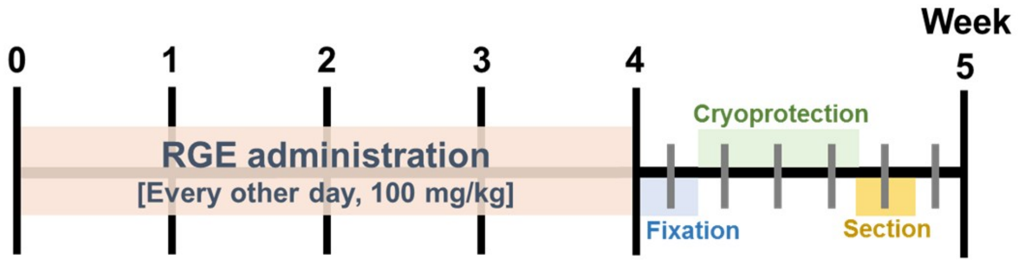

Supplementary Figure 2. The experimental design for evaluation of RGE in 5XFAD mice. Oral administration was performed for treatment with vehicle or RGE every other day for 4 weeks. At 24 h after the last administration, WT and 5XFAD mice were sacrificed and brain sections were analyzed by histological staining.
